# Improvement of Parent’s awareness, knowledge, perception, and acceptability of human papillomavirus vaccination after a structured-educational intervention

**DOI:** 10.1186/s12889-020-09962-1

**Published:** 2020-12-01

**Authors:** Mei Neni Sitaresmi, Nisrina Maulida Rozanti, Lamria Besty Simangunsong, Abdul Wahab

**Affiliations:** 1 Department of Pediatrics, Faculty of Medicine, Public Health, and Nursing, Universitas Gadjah Mada/ DR. Sardjito Hospital, Jalan Gejayan CT X no 18 Pelemkecut, Yogyakarta, 55281 Indonesia; 2 School of Medicine, Faculty of Medicine, Public Health, and Nursing, Universitas Gadjah Mada, Yogyakarta, Indonesia; 3 Department of Biostatistics, Epidemiology and Population Health, Faculty of Medicine, Public Health, and Nursing, Universitas Gadjah Mada, Yogyakarta, Indonesia

**Keywords:** HPV, Cervical cancer, Vaccination, Acceptability, Parents

## Abstract

**Background:**

Regardless of the disease burden of human papillomavirus (HPV), the vaccine has not been included in the Indonesia National Immunization Program. Since 2017 there was a demonstration program of the HPV vaccination in Yogyakarta Province. This vaccine was given free to female primary school students in the 5th and 6th grades (11–13 years old). This study aimed to assess whether a structured-educational intervention focus on HPV increases the parental awareness, knowledge, and perceptions toward HPV and the vaccine acceptability.

**Methods:**

We conducted a pre-post structured-educational intervention study from July to August 2017 before the implementation of the HPV vaccination demonstration program, in Kulon Progo District, Yogyakarta Province, Indonesia. Parents of female primary school students grades 5th and 6th were selected using a school-based proportional random sampling. A pediatric resident provided a structured-educational intervention, which consists of the burden and risk of HPV disease, as well as the benefit and safety of the vaccine. Parents were required to complete validated self-administered questionnaires before and after the structured-educational intervention.

**Results:**

A total of 506 parents participated. Before receiving the structured-educational intervention, parents’ awareness of HPV infection and the vaccines were low. Only 49.2% of parents had heard HPV infection, and 48.8% had heard about the vaccine. After the structured-educational intervention, there were significant improvements in parent’s awareness, knowledge, and perceptions of HPV infection, cervical cancer, and HPV vaccination (all *p* < 0.001). HPV vaccine’s acceptability increased from 74.3 to 87.4% (*p* < 0.001). There was a significant correlation between increasing HPV vaccine acceptability with the improvement of awareness, knowledge, and perception toward HPV infection, cervical cancer and HPV vaccination (r = 0.32 to 0.53, *p* < 0.001). After the structured-educational intervention, better knowledge and positive perceptions of HPV vaccination were predictive of HPV vaccine’s acceptability with OR 1.90 (95%CI:1.40–2.57) and OR 1.31(95%CI,1.05–1.63), respectively.

**Conclusions:**

A structured-educational intervention may improve parental awareness, knowledge, and perceptions toward HPV and the acceptability of the vaccine. Further study, a randomized control trial with longer follow-up are needed to evaluate the long-term and actual effectiveness of improving parents’ knowledge, perceptions and HPV vaccine acceptability*.*

## Background

Globally, cervical cancer ranks the third most common cancer in women, with a total estimated 569,847 diagnosed cases of invasive cervical carcinoma with 311,365 deaths from the disease annually [1]. In Indonesia, cervical cancer is the second most common cancer and the leading cause of death among women aged 15–40 years, with about 32,469 newly diagnosed cases annually [2]. Cervical cancer is linked to Human PapillomaVirus (HPV) infection [3]. The World Health Organization (WHO) recommended introducing HPV vaccination as part of a coordinated and comprehensive strategy to prevent cervical cancer and other diseases caused by HPV [4].

As one proactive way to increase HPV vaccine coverage, the Global Alliance for Vaccines and Immunization (GAVI) supports low-middle income countries in Southeast Asia, including Indonesia, to conduct the HPV Demonstration Program [5]. ﻿ The HPV vaccine is added free of charge to an existing school childhood immunization program to girls in primary school, with the 1st dose given for grade 5 (11–12 years old) and the 2nd dose for grade 6 (12–13 years old). This program was initiated in the provinces where the burden of cervical cancer cases was the highest and the provinces were ready to implement the HPV vaccination program. The first place was in Jakarta Special Province (October 2016), and this effort was continued in the Special Region of Yogyakarta Province (2017), Surabaya (2018), Makassar (2019), and Manado (2019). If the program is successful, then it will be an initial step to add the HPV vaccine into the National Immunization Program (NIP). The WHO recommended 2-doses of HPV vaccine, explaining ﻿that a 2-doses HPV vaccine schedule provides satisfactory immunological outcomes in adolescent girls less than 15 years old [6].

Because children in this age need parental consent for the vaccination, the acceptance of the HPV vaccine is highly dependent on the knowledge, perceptions, and approval of their parents [7, 8], [9]. A systematic review found several factors associated with parents’ acceptance of HPV vaccination for their children including: perceived susceptibility to HPV infection, benefits of vaccination, the safety of the vaccine, social-environmental factors (e.g., social norms, media influence, doctor’s recommendation), and parental factors (educational level, household income, expenses, and attitudes) [10].

The WHO recommended ﻿countries which are introducing the HPV vaccine into their NIP should invest in a comprehensive communication plan to first build community awareness and acceptance for the vaccine and the program. This plan includes ﻿clear program and communication objectives, ﻿understanding community knowledge, attitudes and practices, and ﻿defined target audiences. Communication efforts should reach all key target audiences, especially parents [11]. Parents’ misperceptions and concerns about the vaccine may affect their acceptance and coverage of the vaccine [12]. Inadequate knowledge is one of the significant obstacles to increase HPV vaccine acceptability [13, 14]. Prior studies in Indonesia reported that parents had a poor baseline knowledge about HPV infection, cervical cancer, and the HPV vaccine [15, 16]. Educating parents was demonstrated to be an effective method to improve vaccine acceptability by increasing their knowledge and positive perceptions toward vaccination. A school-based HPV vaccination education program for girls 9–14 years old and their parents in Hongkong, resulting in higher uptake of HPV vaccine [17]. ﻿A hospital-based, brief, HPV-focused intervention improved the awareness and acceptability of HPV vaccine among ﻿rural Chinese women’s [18]. Another study in China assessing ﻿a short education seminar designed for low educational caregivers showed a remarkable improvement in vaccination knowledge [19].

To our knowledge, no study in Indonesia has assessed the impact of a structured-educational intervention on parental knowledge, perceptions, and acceptability of HPV vaccination. This study aimed to evaluate whether a structured-educational intervention about HPV risks and the HPV vaccine increases the parental awareness, knowledge, and perceptions regarding HPV and the HPV vaccination acceptability.

## Methods

We conducted a one group pre-test post-test design study in KulonProgo District, one of districts in Yogyakarta Province, Indonesia, where the demonstration program was implemented. The study was conducted from July to August 2017, before the implementation of the HPV vaccination demonstration program. This research was part of a larger study assessing knowledge, perceptions, and acceptability of parents and teachers regarding HPV related to the disease and the vaccine. Parents of female primary school students grades 5th (11–12 years old) and 6th (12–13 years old) were selected using a school-based proportional random sampling. A study information statement was sent to the parents, and they were invited to participate in the study. We excluded parents who cannot read and write.

After collecting socio-demographic and baseline data on knowledge, perceptions, and acceptability on HPV infection and the vaccine, a structured-educational intervention was provided to groups of parents in the classroom. Each group consisted of 40–50 parents. The researcher developed the intervention based on literature review and using the framework of the health belief model (HBM), ﻿which emphasize that a person’s perception, such as the perception of disease severity, susceptibility, barriers and benefits, influence the intention and actual health-seeking behavior [20]. The intervention consists of a PowerPoint (PPT) presentation provided by a trained pediatric resident, ﻿in simple understandable language; followed by an interactive discussion. The session lasted around an hour. The presentation explained the burden and risk of HPV disease, as well as the benefit and safety of HPV vaccination, including common misconceptions. The post-intervention data were collected immediately after the educational intervention. Team researcher developed the questioner based on literature review and using the framework of the HBM. ﻿The face and content validity of the questioner was made by pediatrician who experts in the field. The questioner was piloting to 30 parents who have similar characteristics of the study population. We assessed reliability by the internal consistency of the questionnaire reporting Cronbach’s alpha coefficient of: subscale knowledge of HPV infection was 0.9; knowledge of cervical cancer was 0.7, knowledge of HPV vaccination was 0.8, perception of HPV infection was 0.7; perception of cervical cancer was 0.7 and perception on HPV vaccination was 0.8.

Data were collected using validated self-administered questionnaires, which consisted of awareness (4 questions), knowledge and perceptions about HPV infection (14 and 6 questions), knowledge and perceptions about cervical cancer (3 and 2 questions), and knowledge and perceptions about HPV vaccination (5 and 2 questions). For the assessment of knowledge scores, if the answer was correct then it was given a value of 1 and if the answer was incorrect then a value of 0. The total score of awareness ranged from 0 to 4, the score of knowledge on HPV infection ranged from 0 to 14, the score of knowledge on cervical cancer ranged from 0 to 3, and the score of knowledge on HPV vaccine ranged from 0 to 5. Questions about perceptions used a 5-point Likert scale, ranging from strongly disagree (was given value of 1) to strongly agree (was given value of 5). The total score of perception on HPV infection ranged from 6 to 30, the score of perception on cervical cancer ranged from 2 to 10, and the score of perception on HPV vaccine ranged from 2 to 10. Acceptability ﻿was measured by a dichotomous item asking whether they are willing their daughters to get the HPV vaccine, free of charge (yes or no answer options). The full item questioner was presented in supplementation File (Table 6). Ethical approval was attained from the Ethics Committee of the Faculty of Medicine, Public Health and Nursing, Universitas Gadjah Mada, Yogyakarta, Indonesia (KE/FK/0815/EC/2017). We obtained informed consent from all.

participants. Participation was voluntary, and confidentially was insured.

Descriptive statistics were used to describe the distribution, frequency, and percentage of variables. The statistical significance of the pre−/post- test intervention was assessed using McNemar and Wilcoxon Signed-Rank tests. Logistic regression analysis was used to determine the associations between potential predictors and vaccine acceptability. All analyses were performed with two-sided tests, and a value of *p* < 0.05 was considered significant.

## Results

Of the 546 parents who were invited, 513 parents attended the structured-educational intervention (response rate was 94%), seven parents were excluded due to inability to read and write, resulting in a total data set of 506 participants. The majority of the respondents are mothers (92.8%), with a median age of 40 years, and the majority are Moslem (98.2%). Forty-three participants (8.8%) experienced a history of any type of cancer in their family, and 4.4% mentioned that their daughter had already received the HPV vaccination (Table 1).

**Table 1:** Characteristics of respondents

	N	%
Characteristics		
Gender (*n* = 497)		
Male	36	7,2
Female	461	92,8
Age (*n* = 488)		
< 45 years old	342	70,1
> 45 years old	146	29,9
Religion (*n* = 498)		
Muslim	489	98,2
Non-Muslim	9	1,8
Education (*n* = 494)		
Basic education	222	44,9
Higher education	272	55,1
Employment Status (*n* = 506)		
Employed	343	67,8
Unemployed	163	32,2
Health insurance (n = 506)		
Yes	385	76,1
No	121	23,9
Number of children in family (n = 488)		
< =2	79	16,2
> 2	409	83,8
monthly Expense (*n* = 483)		
> 1,500,000 (> 125 US $)	264	54,7
< =1,500,000 (< 125 US $)	219	45,3
History of any cancer in the family (n = 488)		
Yes	43	8,8
No	445	91,2

### Awareness, knowledge, and perceptions of HPV infection, cervical cancer, and HPV vaccination before the structured-educational intervention

The majority of the parents had heard about sexually transmitted infections (STIs) (72.7%) and cervical cancer (73.7). However, only 46.2% of them had ever heard about HPV infection, and 46.2% of them mentioned that HPV caused cervical cancer. The majority (76.5%) of respondents already knew that the vaccination could prevent infection, but only 44.1% of parents had ever heard of the HPV vaccination (Table 2).

**Table 2:** Parent’s knowledge before and after intervention program

Awareness and knowledge	Correct response		
	Before	After	*p*
	n (%)	n (%)	
**Awareness**			
Heard STIs (Yes)	368 (72.7)	404 (79.8)	< 0.001
Heard HPV infection (Yes)	234 (46.2)	349 (69.0)	< 0.001
Heard Ca cervix (Yes)	373 (73.7)	411 (81.2)	< 0.001
Heard HPV vaccine (Yes)	223 (44.1)	354 (70.0)	< 0.001
**Knowledge of STIs**			
Included in STIs			
HPV (Yes)	142 (35.2)	317 (78.7)	< 0.001
HIV (Yes)	261 (64.4)	338 (83.5)	< 0.001
Syphilis (Yes)	152 (40.0)	284 (74.7)	< 0.001
Gonorrhea (Yes)	84 (22.6)	237 (63.9)	< 0.001
Herpes simplex virus (Yes)	104 (27.9)	261 (70.0)	< 0.001
Causes of STIs			
Having sex with STIs patient (Yes)	287 (66.6)	368 (85.4)	< 0.001
Kissing with STIs patient (Yes)	83 (20.9)	45 (11.3)	< 0.001
Swimming in the pool with STIs patient (No)	97 (25.8)	255 (67.8)	< 0.001
Hugging with STIs patient (No)	131 (34.6)	259 (68.3)	< 0.001
HPV-related diseases			
Cervical cancer (Yes)	192 (45.0)	345 (80.8)	< 0.001
Genital warts (Yes)	96 (24.6)	285 (72.9)	< 0.001
Oral cancer (Yes)	83 (21.2)	286 (73.1)	< 0.001
Urinary Tract Infection (UTIs) (No)	64 (17.1)	160 (42.8)	< 0.001
Bladder cancer (No)	46 (12.3)	104 (27.7)	< 0.001
**Knowledge about Cervical Cancer**			
Keep clean enviroment can reduce risk of Ca Cervic (No)	76 (17.8)	177 (41.5)	< 0.001
Cervical cancer can occur in women and men (No)	183 (42.7)	216 (50.3)	< 0.001
Cervical cancer is caused by HPV infection (Yes)	235 (55.3)	359 (84.5)	< 0.001
**Knowledge about HPV Vaccination**			
Vaccination is one way to prevent infections (Yes)	325 (76.5)	379 (89.2)	< 0.001
HPV vaccine does not give protection from cervical cancer (No)	174 (42.1)	285 (69.0	< 0.001
HPV vaccine can be given to women and men (Yes)	87 (22.0)	261 (65.9)	< 0.001
HPV vaccine is given to children (No)	58 (17.1)	142 (41.8)	< 0.001
HPV vaccine is given to Adolescent (Yes)	224 (58.5)	326 (85.1)	< 0.001

At baseline, the majority of parents already have a strong perception of the severity of cervical cancer. Most parents were “agree/strongly agree” that cervical cancer is a dangerous cancer (87.8%), and every woman was at risk of having cervical cancer (72.6%). However, the perception of the risk of having HPV infection was low, with only 40.3% strongly agree or agree that they are at risk of having HPV infection. The perceptions of the benefit and safety of the HPV vaccine were high; 78.6% of respondents strongly agree/agree that they believe HPV vaccination is useful for preventing cervical cancer, and 69.7% of them strongly agree/agree that they believe HPV vaccine is safe (Table 3).

**Table 3:** Parent’s perception before and after intervention program

Perception	agree/strongly agree		
	Before n (%)	After n (%)	*p*
**Perception toward HPV infection**			
HPV infection is a common STis in women	266 (57.7)	368 (80.5)	< 0.001
All person, both women and men are at risk for having HPV infection	186 (40.3)	365 (81.8)	< 0.001
HPV infection can cause cervical cancer	280 (61.5)	381 (84.3)	< 0.001
HPV infection is a mayor cause of women death	276 (61.5)	362 (81)	< 0.001
Behavioural can prevent HPV infection			
No sex before marriage	420 (88.2)	430 (94.7)	< 0.001
HPV vaccination	385 (84.8)	411 (92.6)	< 0.001
**Perception toward cervical cancer**			
Cervical cancer is a dangerous cancer	416 (87.8)	437 (95.8)	< 0.001
Every women is at risk of developing cervical cancer	337 (72.6)	394 (86.6)	< 0.001
**Perception toward HPV vaccination**			
I believe HPV vaccination is useful for preventing cervical cancer	367 (78.6)	415 (92.7)	< 0.001
I believe HPV vaccine is safe	317 (69.7)	402 (91.0)	< 0.001

### Factors associated with HPV vaccine’s acceptability after the structured-educational intervention

In the bivariate analysis, we found that knowledge and perceptions of HPV infection, cervical cancer, and HPV vaccine were predictive of vaccine acceptability. There was no correlation between the sociodemographic characteristics of the respondents with HPV vaccination acceptability. In the multivariate analysis, we found that better knowledge of HPV vaccination and more positive perceptions of HPV vaccine were predictive of vaccine acceptability with OR 1.90 (95%CI:1.40–2.57) and OR 1.31(95%CI:1.05–1.63), respectively (Table 4).

**Table 4:** Bivariate and multivariate analysis of awareness, knowledge, perception and acceptability of HPV vaccine: after intervention program

Analysis	Bivariate analysis		Multivariate analysis	
	OR (95% CI)	*p*	OR (95% CI)	p
Awareness	1.56 (1.22–2.00)	0.000	0.86 (0.60–1.25)	0.448
Knowledge of STIs	1.21 (1.13–1.29)	0.000	1.05 (0.92–1.12)	0.479
Knowledge about Cervical Cancer	2.09 (1.48–2.96)	0.000	0.78 (0.94–0.59)	0,787
Knowledge about HPV Vaccination	2.70 (1.70–2.53)	0.000	1.90 (1.40–2.57)	0.000
Perception about HPV infection	1.13 (1.07–1.18)	0.000	0.97 (0.89–1.06)	0.599
Perception about cervical cancer	1.30 (1.11–1.52)	0.000	0.93 (0.75–1.15)	0.525
Perception about HPV vaccination	1.62 (1.35–1.92)	0.000	1.31 (1.05–1.63)	0.016

Considerations for accepting HPV vaccinations were free of charge (according to 95.5% of respondents), the vaccine was included in NIP (according to 97.1% of respondents), the vaccine is considered safe by Moslem standards or *halal* (98.0%), scientifically safe (97.0) and effective in preventing the disease (97.7%). The three most important considerations were free of charge, the vaccine is *halal*, and the vaccine is effective.

### Impact of the structured-educational intervention

After the structured-educational intervention, there were significant improvements in parents’ awareness, knowledge, and perceptions of HPV infection, cervical cancer, and the vaccine, all *p* < 0.001(Table 5). Parental acceptability of the HPV vaccination for their children increased significantly (from 74.3 to 87.4%; *p* < 0.001). There was a significant correlation between increasing vaccine acceptability with improvement of awareness (r = 0.33, *p* < 0.001), knowledge of HPV infection (r = 0.35, *p* < 0.001), cervical cancer (r = 0.35, *p* < 0.001) and HPV vaccination (r = 0.47, *p* < 0.001) and perceptions of the HPV infection (r = 0.36, *p* < 0.001), cervical cancer (r = 0.35, *p* < 0.001) and HPV vaccination (r = 0.53, *p* < 0.001).

**Table 5:** Total score of parent’s awareness, knowledge and perception before and after structured-educational intervention

		Mean (SD)	Median	*p*
Awareness	Before	2.36 (1.21)	2	< 0.001
	After	3.00 (1.24)	4	
Knowledge of HPV infection	Before	4.11 (3.80)	3	< 0.001
	After	7.65 (4.64)	10	
Perception of HPV infection	Before	22.80 (7.03)	24	< 0.001
	After	24.79 (8.88)	27	
Knowledge of cervical cancer	Before	1.14 (0.95)	1	< 0.001
	After	1.51 (0.99)	2	
Perception of cervical cancer	Before	7.67 (2.47)	8	< 0.001
	After	8.03 (2.93)	9	
Knowledge of HPV vaccination	Before	1.95 (1.48)	2	< 0.001
	After	3.05 (1.72)	3	
Perception of HPV vaccination	Before	7.31 (2.57)	8	< 0.001
	After	7.81 (3.07)	9	

Score of awareness ranged from 0 to 4, knowledge of HPV infection ranged from 0 to 14, perception of HPV infection ranged from 6 to 30, knowledge of cervical cancer ranged from 0 to 3, Perception of cervical cancer ranged from 2 to 10, knowledge of HPV infection ranged from 0 to 5, perception of HPV infection ranged 2–10

## Discussion

This research was a school-based study to assess whether a structured-educational intervention can improve parental awareness, knowledge, and perceptions about HPV infection, cervical cancer, and HPV vaccination as well as acceptability of HPV vaccination. Before the intervention, most of the parents had poor baseline knowledge and some negative perceptions of HPV. After the intervention, there was a significant improvement in parents’ HPV-related knowledge and perceptions as well as their intention to permit the HPV vaccine for their children.

At the baseline, we found that most of our participants had heard about STIs and cervical cancer, but less than half of them had heard about HPV infection and the vaccine. A previous qualitative study assessing parental knowledge and perceptions of HPV and cervical cancer prevention in rural Central Java, Indonesia (2015) found that most respondents have limited knowledge of HPV and the vaccine as well as the relationship between HPV and cervical cancer [15]. Compared to a study 4-years earlier which was conducted in the same province (Yogyakarta Province) in 2013, there was almost no improvement in parental awareness of HPV vaccination (44% of our respondents compared to 47% of earliest study respondents had heard availability of the HPV vaccination), and the percentage of parents who reported their children had received the vaccine (8.0% of our respondents compare to 4.4% of earliest study respondents reported their children had received the vaccine) [16]. Since the vaccine has not been included in Indonesia NIP then this may be the reason why the community is not very aware of the availability of the vaccine and this causes the low vaccine uptake. These findings are similar to previous studies in developing countries where HPV vaccination has not been included in their NIP [18, 21].

There were several factors related to the acceptability of HPV vaccination. From the regression analysis, we found that better knowledge and positive perceptions of the HPV vaccination ﻿were significantly associated with higher acceptability. In line with our study, previous reports have shown that parents’ knowledge about HPV was positively correlated with HPV vaccination acceptability [9, 12]. Perceived risks of HPV infection, perceived vaccine benefits, and vaccine safety were associated with the acceptability of HPV vaccination. Perceptions that their children were at greater risk of getting cancer indicated more desire to permit their children to get the HPV vaccine. Parents wanting to protect their children from cervical cancer and other HPV related diseases as the perceived benefits of HPV vaccination, had a strong relationship with the intention to vaccinate. On the other hand, parents who were not sure about HPV vaccine efficacy and afraid of any side effects which might be harmful to their children were less likely to accept the vaccine [22–28]. This finding emphasized the importance of interventions to modify parents’ knowledge, perceptions, and beliefs regarding HPV disease and the vaccines.

We found that besides the vaccine’s benefits and safety, free of charge, the vaccine being included in NIP, and the vaccine considered *halal* ﻿(permissible under Islamic Shariah Law) were important considerations for accepting the vaccine. Including HPV vaccine in the NIP and providing health insurance could eliminate the cost barriers. Because the majority of our study population is Muslim, the *halal* issue is an important consideration for accepting the vaccine. Previously, the non-*halal* status was reported as a barrier to the rotavirus vaccine acceptability in Yogyakarta, Indonesia. Engagement of religious leaders is needed to facilitate the bridge between immunization programs and the community, particularly during the introduction of a new vaccine [29].

Earlier studies found that parents need HPV-related information to decide whether to accept or refuse the HPV vaccination [8, 13, 30]. In line with the WHO recommendations [11], a systematic review of knowledge, attitudes, and barriers toward HPV vaccination in developing economic countries of South-East Asia Region emphasized ﻿the importance of educational campaigns before the vaccine is included in the NIP ﻿to improve vaccine uptake [22]. Parents should be provided with more detailed information about HPV infection, cervical cancer, and HPV vaccination, which would help parents avoid any misunderstandings and to change parents toward a positive attitude.

Previous studies conducted in more developed countries showed the effectiveness of digital media/ website to educate parents. A randomized cluster trial, parents of 11–17 years old girls watched a digital video outlining the risks and benefits of the vaccine was conducted in Indiana, United States. It showed that adolescents whose parents watched the video had a 3-times higher odds of receiving a dose of the HPV vaccine [31].. Another study assessing the effect of a school-based HPV vaccination program used video presentation, and a website for girls 9–14 years old and their parents was conducted in Hongkong. The program resulted in a higher uptake of the HPV vaccine [17].

In developing countries such as Indonesia, which internet access is not always available, providing a more “conventional” education would be more feasible and acceptable. Our study found that a structured-education intervention using a standard PPT presentation followed by an interactive discussion focused on HPV disease and the vaccine improved parents’ knowledge, perception, and acceptability. This result emphasized the effectiveness of “conventional” educational interventions to target consumers/ parents in improving the acceptability of vaccines proven by previous studies. A hospital-based, brief, HPV-focused intervention, using a PPT presentation, improved the awareness and acceptability of HPV vaccine among rural Chinese woman’s [18]. Another study in China assessing a short-education seminar designed for low educational level caregivers showed a remarkable improvement in vaccination knowledge [19]. A one-hour educational presentation was effective in improving Malaysian parent’s knowledge and had an essential impact on vaccine acceptance [32]. A school-based intervention study assessed the effect of an hour PPT presentation intervention on HPV knowledge and attitudes towards HPV and its vaccine among junior school students in Chengdu, China. The study results demonstrated the effectiveness of this intervention in improving HPV knowledge among students and increasing their willingness to be vaccinated [33]. Even though we used a structured PowerPoint presentation, we should consider the presenter’s role in facilitating interactive discussion. Therefore, when implementing this module, we should conduct training for facilitators who will provide this intervention with skill “*how to facilitate an interactive discussion”*.

The structured-educational intervention conducted in our study, in the form of a presentation and interactive discussion by professional health educators, was shown to significantly improve the parents’ knowledge and perceptions about HPV. Giving educational campaigns could increase parental knowledge and perceptions about HPV, concurrently with physician recommendations and nationally funded vaccination programs [21, 28]. A systematic scoping review (2019) regarding communication concerning HPV vaccinations in low and middle-income countries found that physicians were important components to deliver information about HPV and give parents a recommendation to get HPV vaccination for their children [23]^.^ Another systematic review conducted by Radisic et al. (2017) found that uptake of HPV vaccination ﻿may be facilitated by encouraging health care provider endorsement [10].

This research has some limitations. This one group pre-test -post-test design study without a control group did not allow us to confirm any causal relations between the intervention and outcomes. A pre- and post-test without follow-up could not determine the long-term effectiveness of the educational intervention on HPV vaccination acceptability. Further study, a randomized control trial with longer follow-up are needed to evaluate the long-term and actual effectiveness of improving parents’ knowledge and perceptions about the HPV vaccination as well as the vaccine acceptability. The strength of this study is that it assessed the effectiveness of an education intervention before the implementation of the HPV vaccination demonstration program, which included a representative sample of parents in Kulon Progo, a rural District. The results of this study can provide important evidence ﻿to the policy-makers about the importance of health education.

## Conclusions

A structured-educational intervention may improve parental awareness, knowledge, and perceptions toward HPV as well as the acceptability of the vaccine. There was a significant correlation between improving the parental knowledge and perceptions of HPV vaccine with increasing acceptability of the HPV vaccine. ﻿A structured-educational intervention designed for parents may have important implications for improving vaccine acceptability. Further study, a randomized control trial with longer follow-up are needed to evaluate the long-term and actual effectiveness of improving parents’ knowledge, perceptions and HPV vaccine acceptability.

## Supplementary Information

**Additional file 1: Table 6** The full item questioner

## Data Availability

The datasets generated and/or analysed during the current study are not publicly available but are available from the corresponding author on reasonable request.

## Abbreviations

GAVI: Global Alliance for Vaccines and Immunization
HPV: Human PapillomaVirus
PPT: PowerPoint
NIP: National Immunization Program
STIs: Sexually-transmitted Infections
WHO: World Health Organization
