# Supplementary material for: Improvement of Parent’s awareness, knowledge, perception, and acceptability of human papillomavirus vaccination after a structured-educational intervention
Source: BMC Public Health. 2020 Dec 1;20:1836. doi: 10.1186/s12889-020-09962-1 (PMC7708115; doi:10.1186/s12889-020-09962-1)
Supplement: Supplementary file 1 — Additional file 1: Table 6 The full item questioner [file 12889_2020_9962_MOESM1_ESM.docx]

Table 6. The full item questioner

PARENTS DEMOGRAFI

| KODE : [__\|__\|__\|__\|__\|__]  Surveyor : [__\|__\|__] ________________________  Date of Survei : _ _ /_ _ /_ _ _ _  Date of data checking : _ _ /_ _ /_ _ _ _ validated by: [__\|__\|__] _____________________  Date of data entry : _ _ /_ _ /_ _ _ _ Operator : [__\|__\|__] _____________________  Date of final checking : _ _ /_ _ /_ _ _ _ |
| --- |
| Students/ child data  Name :________________________________________________  Age: [__\|__] Years; Gender: M/F School : ___________________(  phone number: _________________________Address : ______________________________ |

| No. | question  *(please fill ( √)* | | | | | | | | |  |  |
| --- | --- | --- | --- | --- | --- | --- | --- | --- | --- | --- | --- |
| Q.4.1 | What is your education level? | | 1. [ ] not finish elementary school 2. [ ] elementary school 3. [ ] Yunior high school 4. [ ] Senior high school 5. [ ] Diploma 6. [ ] university (undergraduate, master, doctoral) 7. [ ] do not want to answer | | | | | | |  |  |
|  |  |  |  |  |  |  |  |  |  |  |  |
| Q.4.2 | What is your occupation? | | | | | | |  |  |  |  |
|  |  | 1. [ ] entrepreneur 2. [ ] full time employee 3. [ ] part time employee 4. [ ] farmer 5. [ ] others 6. [ ] no work 7. [ ] do not want to answer | | | | | | | |  |  |
| Q4.3 | What is your religion? | | | 1. [ ] Muslim 5. [ ] Budha 2. [ ] Katolik 6. [ ] Konghucu 3. [ ] Kristen 7. [ ] other : ____________ 4. [ ] Hindu | | | | | |  |  |
| Q.4.4 | What type of health insurance do you have? | | | | | | 1. [ ] none 2. [ ] insurance paid by government 3. [ ] self-payment government insurance 4. [ ] private insurance 5. [ ] others : _________________ | | |  |  |
| Q.4.5 | Access to health facilities | | | | | | | | |  |  |
|  | 1. How far is your house to the health facilities? | | | |  | | | | | |  |
|  | 1. Primary health center | | | | 1. [ ] < 1 Km 2. [ ] 1-5 Km 3. [ ] > 5 Km | | | | | |  |
|  | 1. Hospitals | | | | 1. [ ] < 1 Km 2. [ ] 1-5 Km 3. [ ] > 5 Km | | | | | |  |
|  | 1. Midwives/ nurses 2. Doctors | | | | 1. [ ] < 1 Km 2. [ ] 1-5 Km 3. [ ] > 5 Km  1. [ ] < 1 Km 2. [ ] 1-5 Km 3. [ ] > 5 Km | | | | | |  |
|  | 1. How easily can you reach the nearest health facility | | | | | 1. [ ] very easy 2. [ ] easy 3. [ ] difficult 4. [ ] very difficult | | | | | |
|  | c. Which of the following is your main daily transportation | | | | | 1. [ ] bicycle 2. [ ] motorcycle 3. [ ] car 4. [ ] public transportations 5. [ ] others:______________ | | | | | |
| Q.4.6 | How many rupiah is your monthly average family expenditure in the last three months? | | | | | 1. [ ] < Rp. 500.000 2. [ ] Rp 500.000 – 1.500.000 3. [ ] Rp. 1.500.000 – 3.000.000 4. [ ] > Rp. 3.000.000 IDR 5. [ ] do not want to answer | | | | | |
| Q.4.7 | How many children do you have? | | | | | 1. [ ] 1 2. [ ] 2 3. [ ] 3 4. [ ] > 3 | | | | | |
| Q.4.8 | Is there a family history of cancer? | | | | | 1. [ ] Yes 2. [ ] No | | | | | |

Part 1

| No. | PERTANYAAN | JAWABAN | |
| --- | --- | --- | --- |
| Q.1.1 | Have you ever heard/ be informed of Sexually Transmitted infection? | 1. [ ] Yes 2. [ ] No | |
| Q.1.2 | Have you ever heard/ be informed of HPV infection? | 1. [ ] Yes 2. [ ] No 🡪 ke Q.1.3 | |
|  | Where did you get this information?  *answers can be more than one* | 1. [ ] Family 2. [ ] health workers 3. [ ] teachers 4. [ ] friend 5. [ ] elektronik media (radio, TV, *website*) 6. [ ] Social Media (facebook, instagram, twitter) 7. [ ] Media (newspaper, bulletin) | |
| Q.1.3 | Which one is Sexually Transmitted Infections (STIs)? | | |
|  | *Human papilloma virus* (HPV) infection | | [ ] right [ ] false [ ] do not know |
|  | *Human immunodeficiency virus* (HIV) infection | | [ ] right [ ] false [ ] do not know |
|  | Sifilis infection | | [ ] right [ ] false [ ] do not know |
|  | Gonorrhoea infection | | [ ] right [ ] false [ ] do not know |
|  | Herpes infection | | [ ] right [ ] false [ ] do not know |
|  | | | |
| Q.1.4 | Which one is the cause of STIs?: | | |
|  | Having sex with STIs patient | | [ ] right [ ] false [ ] do not know |
|  | Swimming in the pool with STIs patient | | [ ] right [ ] false [ ] do not know |
|  | kisssing with STIs patient | | [ ] right [ ] false [ ] do not know |
|  | hugging with STIs patient | | [ ] right [ ] false [ ] do not know |
|  | | | |
| Q.1.5 | This following condition is caused by *Human papilloma virus* (HPV): | | |
|  | cervical cancer | | [ ] right [ ] false [ ] do not know |
|  | genital warts | | [ ] right [ ] false [ ] do not know |
|  | unitary tract infection | | [ ] right [ ] false [ ] do not know |
|  | bladder cancer | | [ ] right [ ] false [ ] do not know |
|  | oral cancer | | [ ] right [ ] false [ ] do not know |
|  |  | |  |

| Q.1.6 | | | What do you perceive regarding these following statements? : | strongly agree | agree | not sure | disagree | | strongly disagree | | |
| --- | --- | --- | --- | --- | --- | --- | --- | --- | --- | --- | --- |
|  | | | HPV infection is a sexual transmitted infection that often occurs in woman | | 1[ ] | 2[ ] | 3[ ] | 4[ ] | | 5[ ] | |
|  |  |  | All person, both woman and man are at risk for having HPV infection | | 1[ ] | 2[ ] | 3[ ] | 4[ ] | | 5[ ] | |
|  |  |  | HPV infection may cause cervical cancer | | 1[ ] | 2[ ] | 3[ ] | 4[ ] | | 5[ ] | |
|  |  |  | HPV infection is a major cause of women death | | 1[ ] | 2[ ] | 3[ ] | 4[ ] | | 5[ ] | |
| Q.1.7 | The following behaviors can prevent HPV infection: | | | strongly agree | agree | not sure | disagree | strongly disagree | |  |  |
|  | Not having sexual intercourse before marriage | | | 1[ ] | 2[ ] | 3[ ] | 4[ ] | 5[ ] | |  |  |
|  | HPV vaccination | | | 1[ ] | 2[ ] | 3[ ] | 4[ ] | 5[ ] | |  |  |

Part 2

| Q.2.1 | Have you ever heard/ be informed of Cervical cancer | 1. [ ] yes 2. [ ] not yet 🡪 Q.2.3 | | | | | |
| --- | --- | --- | --- | --- | --- | --- | --- |
| Q.2.2 | Where did you get this information?  *answers can be more than one* | 1. [ ] Family 2. [ ] health workers 3. [ ] teachers 4. [ ] friend 5. [ ] elektronik media (radio, TV, *website*) 6. [ ] Social Media (facebook, instagram, twitter) 7. [ ] Media (newspaper, bulletin) | | | | | |
| Q.2.3 | Cervical cancer can occur in woman and man | [ ] right [ ] false [ ] do not know | | | | | |
|  | Cervical cancer is caused by HPV infection | [ ] right [ ] false [ ] do not know | | | | | |
|  | Keep clean environment can reduce risk of Ca Cervic | [ ] right [ ] false [ ] do not know | | | | | |
| Q.2.4 | What do you perceve regarding these following statements? | strongly agree | | agree | not sure | disagree | strongly disagree |
|  | Cervical cancer is a dangerous cancer | 1[ ] | | 2[ ] | 3[ ] | 4[ ] | 5[ ] |
|  | Every woman is at risk of developing cervical cancer | 1[ ] | 2[ ] | | 3[ ] | 4[ ] | 5[ ] |

PART 3

| Q.3.1 | Have you ever heard of HPV Vaccination? | | 1. [ ] yes 2. [ ] no🡪 to Q.3.3 | | | | | | | | |  |  |  |
| --- | --- | --- | --- | --- | --- | --- | --- | --- | --- | --- | --- | --- | --- | --- |
| Q.3.2 | Where did you get this information?  *answers can be more than one* | | 1. [ ] Family 2. [ ] health workers 3. [ ] teachers 4. [ ] friend 5. [ ] elektronik media (radio, TV, *website*) 6. [ ] Social Media (facebook, instagram, twitter) 7. [ ] Media (newspaper, bulletin) | | | | | | | | |  |  |  |
| Q.3.3 | Vaccination is one way to prevent infections | | [ ] right [ ] false [ ] do not know | | | | | | | | |  |  |  |
|  | HPV vaccine can be given to woman and man | | [ ] right [ ] false [ ] do not know | | | | | | | | |  |  |  |
|  | HPV vaccine does not give protection from Ca cancer | | [ ] right [ ] false [ ] do not know | | | | | | | | |  |  |  |
|  | HPV vaccine is given to: | |  | | | | | | | | |  |  |  |
|  | - children | | [ ] right [ ] false [ ] do not know | | | | | | | | |  |  |  |
|  | - adolescent | | [ ] right [ ] false [ ] do not know | | | | | | | | |  |  |  |
|  | | | | | | | | | | | |  |  |  |
| Q.3.4 |  | | strongly agree | | agree | | not sure | | disagree | | strongly disagree |  |  |  |
|  | I believe that HPV vaccination is useful for preventing cervical cancer | | 1[ ] | | 2[ ] | | 3[ ] | | 4[ ] | | 5[ ] |  |  |  |
|  | I believe that HPV vaccine is safe | | 1[ ] | | 2[ ] | | 3[ ] | | 4[ ] | | 5[ ] |  |  |  |
| Q.3.5 | Has your child received HPV vaccine? | | 1. [ ] yes 2. [ ] not yet | | | | | | | | |  |  |  |
| Q.3.6 | There will be a free HPV vaccination for grade 5 female students as a part of school vaccination program.  Are you willing to have your child get the HPV vaccination? | | 1. [ ] Yes🡪 Q.3.8 2. [ ] No🡪 Q.3.7 3. [ ] unsure/ doubt🡪 Q.3.7 | | | | | | | | |  |  |  |
| Q.3.7 | What are the reasons that you do not allow your child receive the HPV vaccine? or you are still unsure?  (the reasons may be more than one) | | | | | | | | | | |  |  |  |
|  | 1. [ ] I want to discuss with the health workers 2. [ ] I want to receive more information regarding the HPV vaccines 3. [ ] I am afraid of the side effects 4. [ ] I do not believe that the HPV vaccine can protect the disease 5. [ ] I do not believe vaccination 6. [ ] others : ____________________ | | | | | | | | | | | |  |  |
| Q.3.8 | How important is this condition for considering in deciding to get the HPV vaccination? | very important | | important | | doubt | | not important | | very unimportant | | | | |
|  | 1. The vaccine is free | 1 [ ] | | 2 [ ] | | 3 [ ] | | 4 [ ] | | 5 [ ] | | | | |
|  | 1. The vaccine includes in National Program on Immunization | 1 [ ] | | 2 [ ] | | 3 [ ] | | 4 [ ] | | 5 [ ] | | | | |
|  | 1. The vaccine is recommended by Indonesia pediatric association | 1 [ ] | | 2 [ ] | | 3 [ ] | | 4 [ ] | | 5 [ ] | | | | |
|  | 1. The vaccine is recommended by doctors | 1 [ ] | | 2 [ ] | | 3 [ ] | | 4 [ ] | | 5 [ ] | | | | |
|  | 1. Many children have already received the vaccine | 1 [ ] | | 2 [ ] | | 3 [ ] | | 4 [ ] | | 5 [ ] | | | |  |
|  | 1. The vaccine is Halal | 1 [ ] | | 2 [ ] | | 3 [ ] | | 4 [ ] | | 5 [ ] | | |  |  |
|  | 1. Do not violate my religious | 1 [ ] | | 2 [ ] | | 3 [ ] | | 4 [ ] | | 5 [ ] | | |  |  |
|  | 1. The vaccine has already implemented in other countries | 1 [ ] | | 2 [ ] | | 3 [ ] | | 4 [ ] | | 5 [ ] | | |  |  |
|  | 1. The vaccine is safe | 1 [ ] | | 2 [ ] | | 3 [ ] | | 4 [ ] | | 5 [ ] | | |  |  |
|  | 1. The vaccine can protect infection and cancer | 1 [ ] | | 2 [ ] | | 3 [ ] | | 4 [ ] | | 5 [ ] | | |  |  |
|  | 1. Information regarding the vaccine can easily be obtained from heath workers. | 1 [ ] | | 2 [ ] | | 3 [ ] | | 4 [ ] | | 5 [ ] | | |  |  |
|  | 1. The vaccine can easily be accessed | 1 [ ] | | 2 [ ] | | 3 [ ] | | 4 [ ] | | 5 [ ] | | |  |  |
| Based on the above statements, choose the 3 most important reasons | | | | number: [ ], [ ], and [ ] | | | | | | | | |  |  |
| Q.3.9 | Regarding the vaccination, who will you follow their advice? | | | 1. [ ] Doctor 2. [ ] midwifes/ nurses 3. [ ] health volunteers 4. [ ] family/ friends 5. [ ] others:________________ | | | | | | | | |  |  |
